# A Mathematical Model of the Expansion Evolution of Magnesium Oxide in Mass Concrete Based on Hydration Characteristics

**DOI:** 10.3390/ma14123162

**Published:** 2021-06-08

**Authors:** Chuqiao Feng, Cheng Zhao, Xiaomin Yu, Jie Xiong, Longwen Tang

**Affiliations:** 1 Guizhou Engineering Technology Research Center for Exploitation and Utilization of Water Resources in Karst Region, Guiyang 550002, China; fcq0418@163.com (C.F.); yxm@whu.edu.cn (X.Y.); bearxj@263.net (J.X.); 2 State Key Laboratory of Water Resources and Hydropower Engineering Science, Wuhan University, Wuhan 430072, China; 3 Physics of AmoRphous and Inorganic Solids Laboratory (PARISlab), Department of Civil and Environmental Engineering, University of California, Los Angeles, CA 90095, USA; whutang@ucla.edu

**Keywords:** magnesium oxide concrete, expansion, autogenous deformation, mathematical model

## Abstract

The low swelling property of magnesium oxide concrete is a significant feature that can be used to control the cracking of mass concrete. Based on the characteristics of the chemical reaction, this work proposes a coupled hydro-thermo-mechanical model that can be implemented with the finite element method for predicting the autogenous volumetric deformation of magnesium concrete. By introducing the degree of the hydration reaction of magnesia and the degree of the hydration reaction of cementitious materials as intermediate variables of the chemical reaction system, a prediction model of the concrete temperature and chemical fields is established, and using this model, the effect of the temperature on the reaction rate can be considered in real time. In addition, by combining the relationship between the degree of the hydration reaction of magnesium oxide and the comprehensive expansion of concrete, a mathematical model for calculating the expansion stress of magnesia concrete was established. The algorithms were derived by mathematical equations, and the simulation results were compared to the experimental temperature and autogenous volumetric strain curves, which showed that the hydration model provides a relatively high accuracy. The model was also applied to an arch dam, and the coupled thermo-chemical-mechanical responses of mass concrete during construction were investigated. Simulation results show that the increase in temperature (hydration of cementitious material) and expansion volumetric deformation (hydration of MgO) of the concrete on the upstream and downstream surfaces lags obviously behind that of the inner regions. Quantitative analysis for differences of internal and external expansion is worthy of further attention and study on a basis of further experimental data as well as monitored data.

## 1. Introduction

For mass concrete structures, thermal stresses that may induce cracking are a major concern, and temperature control measures must be taken during the construction period. For hydraulic mass concrete structures, cracks are mainly caused by temperature deformation shortly after construction [1]. This early cracking behavior of concrete is essentially the result of the trade-off between strength and stress during the hardening process. The properties of concrete change with age, and the strength gradually increases, which causes the concrete to release a large amount of heat [2]. Under the combined action of the heat of hydration and the cooling of the external environment, a temperature gradient is generated inside the concrete structure.

In the past, to prevent cracks induced by thermal stresses, mass concrete structures were usually poured in layers to artificially increase the construction interval. Moreover, other temperature measures, such as the use of ice to precool aggregates, embedded cooling water pipes, and surface heat preservation, have been used to prevent temperature-induced cracks [3]. However, these measures are not economical, and some measures, such as pipe cooling, are very complex and may seriously affect the construction schedule.

In research and practice, during the dam concrete preparation process, an appropriate amount of magnesium oxide that has been burned under high temperatures can be added to make the concrete expand, which can compensate for shrinkage deformation [4,5] during a temperature drop. In combination with regular auxiliary measures (e.g., surface heat preservation and maintenance), MgO can be added as an expansion agent for mass concrete to control self-generated volume deformation and thereby reduce and eliminate dam concrete cracks. This method can prevent dam cracks and reduce the need for most traditional concrete temperature control measures (aggregate precooling, ice mixing, embedded cooling water pipe, etc.) [6].

The addition of MgO in mass concrete is a superior technique that can reduce project construction and management costs by simplifying the temperature control measures. However, the expansion mechanism of MgO concrete is very complex [7], not only can the hydration of the hydration of cement and the MgO itself have an impact on the autogenous deformation, but the fly ash in the cementing material can have an important influence on the MgO concrete, as well [8]. Thus, further research is required. Usually, the autogenous volume deformation (expansion) and expansion stress during construction need to be simulated numerically, and appropriate models and reasonable parameter studies are crucial for such simulations. Therefore, a model that properly describes the expansion characteristics of concrete based on material properties is necessary.

Since the 1970s, MgO concrete has been used in a large number of hydraulic engineering projects in China, and a large amount of deformation data has been collected [8]. B. Zhu (2002, 2003) [9,10] proposed a method for calculating the difference between the indoor test results and the measured values for the autogenous volumetric deformation of micro-expansion concrete and established a calculation model of the autogenous volume deformation by considering this difference. Based on engineering test data and relevant laboratory tests, G. Yang et al. (2004) [11] suggested that the autogenous volumetric deformation characteristics of MgO concrete under constant temperature conform to a hyperbolic model. Using simulation techniques, G. Zhang et al. (2002, 2005) [12,13,14] comprehensively analysed the influences of concrete elastic modulus growth, creep degree with age and temperature on the amount of magnesium oxide expansion and studied the influences of curing temperature and dam concrete temperature on the effective compensation. S. Liu et al. (2006) [15] established a mathematical model of the relationships between MgO concrete autogenous volume deformation and MgO content, fly ash content and temperature. P. Xu et al. proposed the equivalent age method for calculating the spontaneous volume deformation of MgO concrete. (2008) [16]. Other models, such as the arctangent curve model of C Chen et al. (2008) [17], have also been proposed.

The establishment of an expansion and deformation model of magnesium oxide concrete is very important, and many studies that have been carried out can serve as references for practical use and research and are conducive to the establishment of a simulation model and the technical development and popularisation of MgO expansion concrete. However, a unified standard model that considers the chemical reactions of MgO concrete has not yet been developed. Although some models can consider the effect of temperature on the expansion characteristics of magnesium oxide, the calculation methods are controversial because they do not consider the nature of the chemical reaction (i.e., the material properties are not directly related to the chemical reaction degree). Therefore, additional research and relevant projects are needed.

In this paper, based on hydration kinetics, a coupled thermo-chemical-mechanical model is proposed for describing the hydration properties of magnesium oxide concrete. In this model, two variables are introduced as the intermediate variables of the chemical reaction system within concrete: the hydration degree of cementitious materials, which determines the hydration heat release, and the hydration degree of magnesium oxide, which determines the autogenous volumetric deformation. In addition, the model is numerically implemented with the finite element method, and the temperature, hydration and stress field within concrete can be simulated. Based on a parameter study, the model is applied in a case study that considers an arch dam to evaluate the coupled thermo-chemical-mechanical responses of mass concrete during construction.

## 2. Coupled Thermo-Chemo-Mechanical Model

### 2.1. Transient Heat Transfer

The transient heat transfer process of concrete can be described as follows:

(1) $$\rho C\frac{\partial T(x,t)}{\partial t}=\lambda_{T}\Delta T(x,t)+\frac{\partial Q}{\partial t}$$

(2) $$\frac{\partial Q}{\partial t}{=Q}_{\infty }\frac{\partial \xi }{\partial t}$$

where ρ, C, $\lambda_{T}$, Δ and $Q_{\infty }$ are the density, volumetric heat capacity, thermal conductivity coefficient, Laplacian operator and final volumetric heat of hydration, respectively. The latent heat release due to concrete hydration is a nonlinear and thermally dependent process.

### 2.2. A Chemical Model for the Hydration Process

According to the Arrhenius law, the time-dependent hydration progress can be described as in Equation (2). In the model, the hydration rate is related to the temperature and the chemical affinity $A_{\xi }(\xi )$:

(3) $$\frac{\partial \xi }{\partial t}=A_{\xi }(\xi )\exp(-\frac{E_{a}}{RT})$$

where $E_{a}$ is the activation energy of the reaction and R is the ideal gas constant. Cervera et al. developed an analytical form of the normalized affinity based on thermodynamics [18]. The free energy of the thermo-chemical system can be divided into three parts: the thermal contribution, the thermo-chemical contribution and the chemical contribution. The chemical contribution in this work will be considered as a quartic function instead of a cubic function. Similar to the strategy presented in [19], a modified form of the chemical affinity can be derived [18,20]:

(4) $$A_{\xi }(\xi )=\beta_{1}(\beta_{2}+\xi )(\xi_{\infty }-\xi )\exp(-\bar{\eta }\frac{\xi }{\xi_{\infty }})$$

where $\beta_{1}$, $\beta_{2}$ and $\beta_{3}$ are material coefficients, $\xi_{\infty }$ is the ultimate hydration degree and $\bar{\eta }$ represents the viscosity due to the microdiffusion of free water through hydrates. These parameters can be calibrated using the experimental results.

### 2.3. Stress–Strain Relationship

Concrete strain mainly includes elastic strain, creep strain, thermal strain and autogenous volumetric strain:

(5) $$\dot{\varepsilon }={\dot{\varepsilon }}_{el}+{\dot{\varepsilon }}_{as}+{\dot{\varepsilon }}_{th}+{\dot{\varepsilon }}_{cr}$$

where $\varepsilon_{el}$, $\varepsilon_{cr}$, $\varepsilon_{th}$ and $\varepsilon_{au}$ are the elastic strain, creep strain, thermal strain and autogenous volumetric strain, respectively.

Accordingly, the stress evolution can be described as follows:

(6) $$\dot{\tilde{\sigma }}=E_{\left(\xi \right)}{\dot{\varepsilon }}_{el}=E_{\left(\xi \right)}\left(\dot{\varepsilon }-{\dot{\varepsilon }}_{as}-{\dot{\varepsilon }}_{th}-{\dot{\varepsilon }}_{cr}\right)$$

### 2.4. Mathematical Model of Expansion Evolution

#### 2.4.1. Expansion Rate

The expansion of magnesium oxide concrete is essentially due to the hydration of magnesium oxide to form magnesium hydroxide, which follows the general rule of chemical reactions: temperature and concentration are the main factors that influence the reaction process. A higher temperature corresponds to a faster reaction, a higher hydration rate of magnesium oxide and more expansion. Meanwhile, the expansion rate is proportional to the magnesium oxide content involved in the reaction (i.e., the concentration in the liquid chemical reaction), which conforms to the kinetic chemical reaction equation (usually an Arrhenius equation).

At time *t*, the hydration rate of magnesium oxide can be expressed as a function of chemical reaction affinity and temperature at that time:

(7) $$\frac{\partial \zeta }{\partial t}={\tilde{A}}_{\zeta }\left(\zeta \right)\exp(-\frac{E_{m}}{RT})$$

where ζ is the degree of hydration of magnesium oxide, *E_m_* is the chemical reaction activation energy and *R* is ideal gas constant.

Similar to the hydration process of cement [19], the chemical reaction affinity of magnesium oxide hydration can be obtained through a thermodynamic derivation:

(8) $${\tilde{A}}_{\zeta }(\zeta )=B_{1}(\frac{B_{2}}{\zeta_{\infty }}+\zeta )(\zeta_{\infty }-\zeta )\exp(-{\bar{\eta }}_{m}\frac{\zeta }{\zeta_{\infty }})$$

where *B*_1_ and *B*_2_ are material constants, ${\bar{\eta }}_{m}$ represents the viscosity due to the micro-diffusion of free water through the hydrates and $\zeta_{\infty }$ represents the ultimate hydration degree of magnesium oxide. The parameters *E_m_*, *B*_1_, *B*_2_, ${\bar{\eta }}_{m}$ and $\zeta_{\infty }$ can be obtained via experimentation. Equation (8) can reasonably reflect the influences of the residual concentration ($\zeta_{\infty }-\zeta$), temperature (*T*) and water diffusion rate ($\exp(-{\bar{\eta }}_{m}\frac{\zeta }{\zeta_{\infty }})$) on the magnesium oxide hydration.

#### 2.4.2. Volumetric Strain

The autogenous volumetric strain of MgO concrete $\varepsilon_{au}$ can be composed of the strain caused by the hydration of the cementing material and that of the magnesium oxide hydrating material. A linear or piecewise linear function can be used to describe the total volumetric strain:

(9) $${\dot{\varepsilon }}_{as}={-(k}_{a}\dot{\xi }+k_{b}\dot{\zeta })I(\xi >\xi_{0})$$

where *k_a_* and *k_b_* are material parameters that can be obtained via experimentation, *I* represents a unit tensor and $\xi_{0}$ represents the threshold value of hydration (corresponding to the hydration degree of final condensation).

## 3. Numerical Implementation of the Thermo-Chemical Model

### 3.1. Hydration of Cementitious Material and Magnesium Oxide

The transient heat transfer equation and hydration equations are listed in (1), (3) and (7); to implement the coupled equations in the finite element method, an implicit algorithm of a backward-Euler temporal integration and a Gaussian quadrature spatial integration scheme is adopted. At time t+δt, where δt is the time increment, these equations can be discretized as follows:

(10) $$\frac{\partial \xi_{{}_{t+\delta t}}}{\partial t}=\frac{\left(\xi_{t+\delta t}-\xi_{t}\right)}{\delta t}$$

(11) $$\frac{\partial T_{t+\delta t}}{\partial t}=\frac{\left(T_{t+\delta t}-T_{t}\right)}{\delta t}$$

(12) $$\frac{\partial \zeta_{t+\delta t}}{\partial t}=\frac{\left(\zeta_{t+\delta t}-\zeta_{t}\right)}{\delta t}$$

Therefore, the hydration equation of the cementitious material can be written as

(13) $$\frac{\left(\xi_{t+\delta t}-\xi_{t}\right)}{\delta t}=A_{\xi }(\xi_{t+\delta t})\exp(-\frac{E_{a}}{RT_{t+\delta t}})$$

(14) $$\xi_{t+\delta t}-\xi_{t}-A_{\xi }(\xi_{t+\delta t})\exp(-\frac{E_{a}}{RT_{t+\delta t}})\delta t=0$$

Similarly, the hydration equation of magnesium oxide can be written as

(15) $$\frac{\left(\zeta_{t+\delta t}-\zeta_{t}\right)}{\delta t}=A_{\zeta }(\zeta_{t+\delta t})\exp(-\frac{E_{a}}{RT_{t+\delta t}})$$

(16) $$\zeta_{t+\delta t}-\zeta_{t}-A_{\zeta }(\zeta_{t+\delta t})\exp(-\frac{E_{a}}{RT_{t+\delta t}})\delta t=0$$

Based on the principle of virtual work, the basic energy balance equation for heat transfer can be written as

(17) $$\int_{V}\rho C\frac{\partial T(x,y,z,t)}{\partial t}dV=\int_{S}\lambda_{T}\Delta T(x,y,z,t)dS+\int_{V}Q_{\infty }\frac{\partial \xi }{\partial t}dV$$

Combining this equation with (10) and (11) gives

(18) $$\int_{V}\rho C\frac{\left(T_{t+\delta t}-T_{t}\right)}{\delta t}dV=\int_{S}\lambda_{T}\Delta T(x,y,z,t)dS+\int_{V}Q_{\infty }\frac{\left(\xi_{t+\delta t}-\xi_{t}\right)}{\delta t}dV$$

(19) $$\frac{T_{t+\delta t}-T_{t}}{\delta t}\int_{V}N^{T}\rho CNdV=-\int_{V}\lambda_{T}\nabla N^{T}\nabla T(x,y,z,t)dV+(\int_{V}N^{T}Q_{\infty }\frac{\left(\xi_{t+\delta t}-\xi_{t}\right)}{\delta t}dV)$$

(20) $$\begin{array}{l} \frac{T_{t+\delta t}-T_{t}}{\delta t}C_{{}_{t+\delta t}}=-K_{{}_{t+\delta t}}T_{t+\delta t}+Q\\ (\frac{1}{\delta t}C+K)T_{t+\delta t}=Q+\frac{1}{\delta t}CT_{t} \end{array}$$

where N is the shape–function matrix, the superscript T indicates the transpose of a vector or matrix, and K and C are the conduction and heat capacity matrix, respectively. At each time increment, the coupled Equations (11) and (20) are used to obtain the hydration and temperature fields. To form the Q matrix, $\xi_{t+\delta t}$ and $\zeta_{t}$ must be determined using Equations (14) and (16); thus, the Newton–Raphson iterative method is adopted. In an implicit time-integration algorithm, the nodal temperatures and hydration degrees of cementitious material and magnesium oxide can be solved as follows:

(1) Input variables: δt, $T_{t}$, $\xi_{t}$, $\zeta_{t}$, $K_{t}$ and $C_{t}$;
(2) Set a proper temperature increment value and calculate $T_{t+\delta t}$;
(3) Use a Newton–Raphson iterative method to obtain the values of $\xi_{t+\delta t}$ and $\zeta_{t+\delta t}$:
  (21) $$\xi_{{}_{t+\delta t}}^{k+1}=\xi_{{}_{t+\delta t}}^{k}-\frac{f(\xi_{{}_{t+\delta t}}^{k})}{f^{’}(\xi_{{}_{t+\delta t}}^{k})}$$
  (22) $$f(\xi_{{}_{t+\delta t}}^{k})=\xi_{{}_{t+\delta t}}^{k}-\xi_{t}-A_{\xi }(\xi_{{}_{t+\delta t}}^{k})\exp(-\frac{E_{a}}{RT_{t+\delta t}})\delta t$$
  (23) $$\zeta_{{}_{t+\delta t}}^{k+1}=\zeta_{{}_{t+\delta t}}^{k}-\frac{f(\zeta_{{}_{t+\delta t}}^{k})}{f^{’}(\zeta_{{}_{t+\delta t}}^{k})}$$
  (24) $$f(\zeta_{{}_{t+\delta t}}^{k})=\zeta_{{}_{t+\delta t}}^{k}-\zeta_{t}-A_{\zeta }(\zeta_{{}_{t+\delta t}}^{k})\exp(-\frac{E_{a}}{RT_{t+\delta t}})\delta t$$
  where *k* is the iteration counter, $f^{’}(\xi_{{}_{t+\delta t}}^{k})$ and $f^{’}(\zeta_{{}_{t+\delta t}}^{k})$ are the derivatives of $f(\xi_{{}_{t+\delta t}}^{k})$ and $f(\zeta_{{}_{t+\delta t}}^{k})$, respectively, $\xi_{{}_{t+\delta t}}^{0}=\xi_{t}$, and $\zeta_{{}_{t+\delta t}}^{0}=\zeta_{t}$; this process continues while $\left|\frac{\xi_{{}_{t+\delta t}}^{k+1}-\xi_{{}_{t+\delta t}}^{k}}{\xi_{{}_{t+\delta t}}^{k+1}}\right|>Tol$ and $\left|\frac{\zeta_{{}_{t+\delta t}}^{k+1}-\zeta_{{}_{t+\delta t}}^{k}}{\zeta_{{}_{t+\delta t}}^{k+1}}\right|>Tol$ (Tol is the tolerance of the iteration).
(4) Calculate the Q, $K_{t+\delta t}$ and $C_{t+\delta t}$ matrices and verify the balance of Equation (20). If a state of equilibrium can be reached for Equation (20), then proceed to step (5); otherwise, return to step (2) and correct the temperature increment.
(5) Output variables $T_{t+\delta t}$, $\xi_{t+\delta t}$, $\zeta_{t+\delta t}$, $K_{t+\delta t}$ and $C_{t+\delta t}$.

### 3.2. Expansion and Stress Evolution of Magnesium Oxide Concrete

Through the above steps, the temperatures and hydration degrees of the cementitious material and magnesium oxide can be calculated at each moment. Combined with the relationship between the mechanical properties and the hydration degree of concrete, the stress and strain of concrete at each moment can be obtained. In fact, this method can calculate the temperature, stresses and hydration degree synchronously. However, considering that the stress results have little effect on temperature and hydration (i.e., the stress field is weakly coupled with the temperature and chemical fields), a sequential coupling method is essentially the same as a synchronous computation.

The relationship between the apparent stress and strain of concrete can be described as follows:

(25) $$\dot{\tilde{\sigma }}=E{\dot{\varepsilon }}_{el}=E\left(\dot{\varepsilon }-{\dot{\varepsilon }}_{as}-{\dot{\varepsilon }}_{th}-{\dot{\varepsilon }}_{cr}\right)$$

E is the elasticity matrix, which can be written as

(26) $$E=E(\xi )\left[\begin{array}{cccccc} 1 & \frac{\mu }{1-\mu } & \frac{\mu }{1-\mu } & 0 & 0 & 0\\ \frac{\mu }{1-\mu } & 1 & \frac{\mu }{1-\mu } & 0 & 0 & 0\\ \frac{\mu }{1-\mu } & \frac{\mu }{1-\mu } & 1 & 0 & 0 & 0\\ 0 & 0 & 0 & \frac{1-2\mu }{2(1+\mu )} & 0 & 0\\ 0 & 0 & 0 & 0 & \frac{1-2\mu }{2(1+\mu )} & 0\\ 0 & 0 & 0 & 0 & 0 & \frac{1-2\mu }{2(1+\mu )} \end{array}\right]$$

where *E(ξ)* is Young’s modulus and *μ* is Poisson’s ratio.

The evolution of Young’s modulus can be described as follows:

(27) $$E_{\left(\xi \right)}={\left(\frac{\xi -\xi_{0}}{1-\xi_{0}}\right)}^{r_{e}}E_{\infty }$$

where $E_{\left(\xi \right)}$ and $E_{\infty }$ are the Young’s moduli at degrees of hydration ξ and $\xi_{\infty }$, respectively, $\xi_{0}$ represents the hydration degree of the final set of concrete (the concrete completely loses its plasticity and the strength begins to increase) and $\xi_{0}$ and $\gamma_{e}$ are parameters that depend on the concrete composition.

The thermal strain of concrete can be written as

(28) $${\dot{\varepsilon }}_{th}=-\alpha \dot{T}I$$

where *α* is the coefficient of linear expansion and *I* is the unit tensor {1,1,1,0,0,0}^T^.

Considering the influence of magnesium oxide hydration on concrete expansion, the strain produced by the autogenous deformation of concrete can be written as

(29) $$\left\{ \begin{array}{l} {\dot{\varepsilon }}_{as}=-k_{b}\dot{\zeta }I(\zeta >\zeta_{0})\\ {\dot{\varepsilon }}_{as}=0\begin{array}{cccc} & & & \left(\zeta \leq \zeta_{0}\right) \end{array} \end{array}\right.$$

The creep strain can be calculated by using solidification theory. Therefore, the calculation of volume deformation, strain and the ultimate apparent stress of magnesium oxide concrete during the hydration process can be completed by using the above steps.

## 4. Validation

### 4.1. Experimental Study of Magnesium Oxide Concrete

To scrutinize the deformation characteristics, which are closely related to the hydration properties, necessary experiments should be conducted to obtain the corresponding thermal and mechanical parameters. In this work, the experimental study comes from the engineering field (the Sanjiang Archdam Projiect in Guizhou, China). The usual test indexes of concrete include compressive strength, split tensile strength, bulk density, elastic modulus, ultimate tensile value, linear expansion coefficient, adiabatic temperature rise, thermal conductivity, specific heat and autogenous volume deformation. For MgO concrete, the low expansive characteristic caused by the hydration of magnesium oxide is significant and complicated.

The autogenous volume deformation can be monitored by using a digital electric bridge. A strainmeter is placed in the center of a concrete specimen that has been poured in a bottle. The height of the concrete cylinder is 10 cm and the radius is 60 cm. The resistance can be measured by the digital electric bridge, which is connected to the wire of the strainmeter, and then, the autogenous volume deformation can be calculated. Because the ambient temperature has a considerable impact on the test results, a thermostatic water tank is used to adjust the curing temperature of the specimens.

#### 4.1.1. Materials and Methods

Special ordinary Portland cement is used for tests which is produced by Guizhou cement plant in China. The purity of magnesium oxide is greater than 90% and it is produced by Haicheng, Liaoning, China. The fly ash is secondary ash produced by Guizhou Thermal Power Plant.

Table 1 summarizes the mass fractions of the components of two types of MgO concrete with a gradation degree of three.

#### 4.1.2. Test Results

The corresponding adiabatic temperature rise of the two types of MgO concrete at different ages are listed in Table 2, and the initial temperature for the adiabatic test is the indoor temperature of 20 °C. Table 3 gives the measured autogenous volume deformation results of the concrete specimen for curing temperatures of 20 °C and 30 °C.

### 4.2. Activation Energy of the Magnesium Oxide Hydration

While the activation energies of the cement hydration of concrete for calculations are plentiful in the literature, they are not easily found for magnesium oxide applications. Moreover, the activation energy of magnesium oxide hydration is closely related to the magnesium oxide production technique; thus, the activation energy values in different studies are quite different. Therefore, in this work, a method that can be used to obtain the activation energy of magnesium oxide hydration within concrete is proposed.

According to Equation (7), for an arbitrary degree of hydration of magnesium oxide (ζ), the expansion rate is related to only the temperature of concrete. Thus, for given curing temperatures, the ratio of the expansion rate is a function of temperature:

(30) $$\begin{array}{l} \frac{\frac{\partial \zeta }{\partial t}(T=T_{1})}{\frac{\partial \zeta }{\partial t}(T=T_{2})}=\frac{{\tilde{A}}_{\zeta }\left(\zeta \right)\exp(-\frac{E_{m}}{RT_{1}})}{{\tilde{A}}_{\zeta }\left(\zeta \right)\exp(-\frac{E_{m}}{RT_{2}})}\\ =\exp\left[-\frac{E_{m}}{R}(\frac{1}{T_{1}}-\frac{1}{T_{2}})\right] \end{array}$$

Therefore, to reach the same level of hydration, the required time is related to the curing temperature as well. Equation (7) can be transformed to

(31) $$d\zeta ={\tilde{A}}_{\zeta }\left(\zeta \right)\exp(-\frac{E_{m}}{RT})dt$$

(32) $$\frac{d\zeta }{{\tilde{A}}_{\zeta }\left(\zeta \right)\exp(-\frac{E_{m}}{RT})}=dt$$

By integrating both sides of this equation,

(33) $$\int \frac{d\zeta }{{\tilde{A}}_{\zeta }\left(\zeta \right)\exp(-\frac{E_{m}}{RT})}=\int dt$$

(34) $$t=\int_{0}^{\zeta }\frac{d\zeta }{{\tilde{A}}_{\zeta }\left(\zeta \right)\exp(-\frac{E_{m}}{RT})}$$

Then, the time required to reach a certain degree of hydration under different temperatures can be described as:

(35) $$t_{1}=\int_{0}^{\zeta }\frac{d\zeta }{{\tilde{A}}_{\zeta }\left(\zeta \right)\exp(-\frac{E_{m}}{RT_{1}})}$$

(36) $$t_{2}=\int_{0}^{\zeta }\frac{d\zeta }{{\tilde{A}}_{\zeta }\left(\zeta \right)\exp(-\frac{E_{m}}{RT_{2}})}$$

(37) $$\frac{t_{1}}{t_{2}}=\frac{\exp(-\frac{E_{m}}{RT_{2}})}{\exp(-\frac{E_{m}}{RT_{1}})}=\exp\left[-\frac{E_{m}}{R}(\frac{1}{T_{2}}-\frac{1}{T_{1}})\right]$$

Ultimately, the chemical reaction activation energy of MgO hydration *E_m_* can be determined:

(38) $$\frac{E_{m}}{R}=\frac{\ln\frac{t_{1}}{t_{2}}}{\frac{1}{T_{1}}-\frac{1}{T_{2}}}$$

According to Equation (38) and combined with the experimental data of the autogenous volume deformation of oxygen magnesia concrete samples at different ages with varying curing temperatures, the activation energy can be obtained by choosing two different curing ages at which the hydration degrees are equivalent. In practice, the measurement of autogenous volume deformation cannot be conducted at every age of the magnesium oxide concrete; therefore, close values may be chosen, and the interpolation method should be used to obtain appropriate estimates of *E_m_*.

### 4.3. Numerical Verification

As discussed in Section 2, the exothermic and autogenous volumetric characteristics are associated with the temperature or hydration degree values. In this section, based on the experimental values, the hydration model in this paper is numerically verified, and the parameters are calibrated. Based on the results of the adiabatic experiments and by adopting the strategy in reference [21], the results of the parameter calibration of the thermo-chemical model that describes the properties of cement hydration are listed in Table 4. Due to a lack of experimental data of the adiabatic temperature rise with different initial temperatures, the reaction activation energy can be set to 41570 J mol^−1^ based on references [22,23], and $E_{a}/R$ is equal to 5000 K.

**Figure 1:**
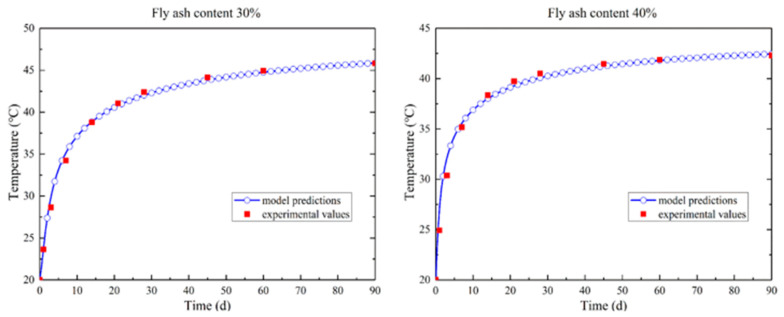
Temperature evolution of magnesium oxide concrete (fly ash content 30% and 40%) under adiabatic conditions.

The temperature evolution of the simulations and the adiabatic tests on the concrete samples with fly ash content 30% and 40% are plotted in Figure 1. The results show that the predictions of the hydration model agree with the experimental values.

Based on the data in Section 4.1 and by adopting the strategy from Section 4.2, the activation energy of the magnesium oxide hydration in concrete can be obtained since the experimental data of autogenous volume deformation of oxygen magnesia concrete samples at different ages with curing temperatures are given. 

Once the activation energy is obtained, the other parameters of the model can be calibrated on the basis of the experimental data of autogenous volume deformation. The results of the parameter calibration of the thermo-chemical model that describes the properties of MgO hydration are listed in Table 5. As shown in the table, given the same MgO content and cement type, the reaction activation energy may vary.

**Figure 2:**
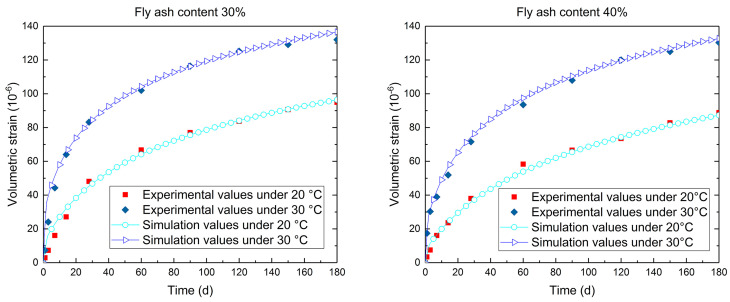
Comparison of the simulation and experimental values of the autogenous volumetric strain of magnesium oxide concrete with various curing temperatures (fly ash content 30% and 40%).

The autogenous volumetric strain evolution of the simulation values and the experimental data for the concrete samples with fly ash content 30% and 40% are plotted in Figure 2. Comparing the simulation values and experimental data, the hydration model proposed in this work shows a good accuracy under different curing temperatures.

### 4.4. Engineering Application

In Section 4.3, the thermo-chemical model parameters are calibrated, and the simulation values show good agreement with the experimental data. In this section, an arch dam in Guizhou, China, was selected for thermo-chemical-mechanical analysis. Thus, the hydration properties and mechanical responses of magnesium oxide in mass concrete can be explored.

**Figure 3:**
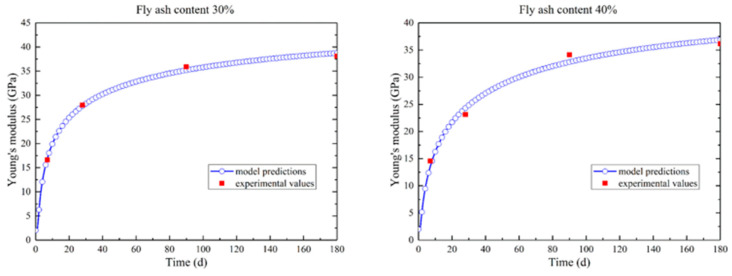
Young’s modulus evolution of magnesium oxide concrete (fly ash content 30% and 40%) under adiabatic conditions.

Mechanical tests on concrete are typically conducted under isothermal conditions; therefore, the hydration process is simulated under isothermal conditions in this research, and the mechanical characteristics can be linked to the hydration degree rather than to time. The evolution of the Young’s modulus for the isothermal test of the fly ash concrete specimens with fly ash content 30% and 40% are plotted in Figure 3. For simplification, Poisson’s ratio is assumed to be constant (0.167).

The Sanjiang Arch Dam, which was constructed with magnesium oxide concrete during 2002 and 2004, was selected as a typical structure for thermo-chemical-mechanical analysis. A birth-and-death technique is adopted to address the existence and nonexistence of the concrete. Corresponding elements in the finite element model are made active when a fresh concrete slab is poured. Thus, the incremental pouring process of concrete can be simulated in the FE model.

For mass concrete structures, thermal stresses are a major concern, and temperature control measures must be taken during construction. There are multiple material and structural factors that impact thermal stress, such as concrete hydration properties, thermal and mechanical boundary conditions, the construction schedule and temperature control measures; for simplification, these conditions are not described in detail. In subsequent studies, these details will be discussed, and the simulation results will be compared with the monitored values.

### 4.5. Results and Discussion

**Figure 4:**
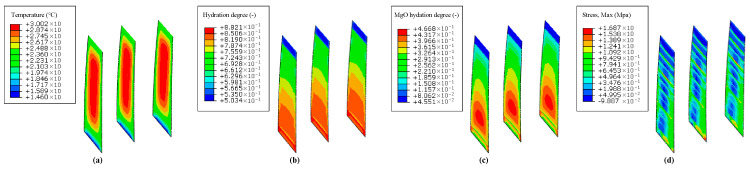
Coupled thermo-chemical-mechanical field computations on 1 March 2003. (**a**) Temperature (°C); (**b**) hydration degree of cementitious material (-); (**c**) hydration degree of magnesium oxide (-); (**d**) first principal stress (MPa).

**Figure 5:**
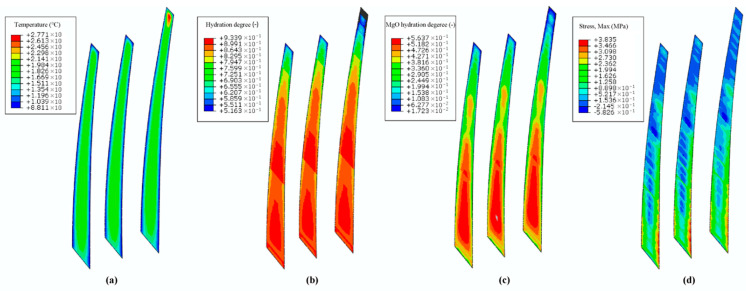
Coupled thermo-chemical-mechanical field computations on 1 June 2003. (**a**) Temperature (°C); (**b**) hydration degree of cementitious material (-); (**c**) hydration degree of magnesium oxide (-); (**d**) first principal stress (MPa).

Based on the parameter studies and numerical validation in Section 4.3 and Section 4.4, temperature and stress fields can be simulated using the hydration model. Figure 4 and Figure 5 show the coupled thermo-chemical-mechanical field simulations on different dates. As shown in Figure 4 and Figure 5, the temperature is uneven within the mass concrete structure, and the hydration degree is also present in an inhomogeneous distribution. For the inner region with poor heat dissipation conditions, the temperature appears to be higher than that of exterior region on the same height. The hydration degree of the cement and that of MgO present the same pattern as the temperature distribution, as well.

**Figure 6:**
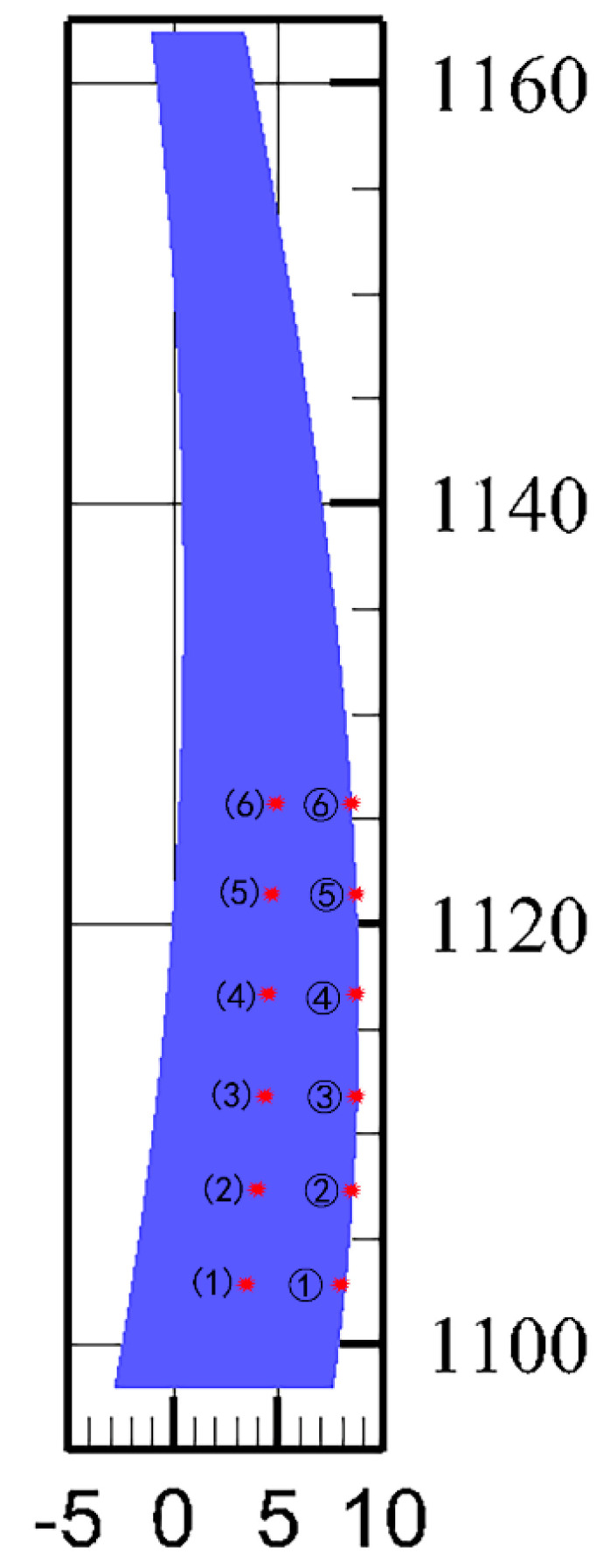
Position distribution of typical points.

The temperature and stress evolution processes are discussed for further comparative analysis. Thus, twelve typical points on the crown cantilever are selected, and points ①, ②, ③, ④, ⑤ and ⑥ are on the upstream profile (exterior areas of the dam), whereas points (1), (2), (3), (4), (5) and (6) are distributed over the midplane (interior areas of the dam). Accordingly, points ① and (1), points ② and (2), points ③ and (3), points ④ and (4), points ⑤ and (5), and points ⑥ and (6) are at six different elevations. The position distribution of the typical points is shown in Figure 6. Thus, the pouring layers of the typical points are different, as is the age of the concrete. 

**Figure 7:**
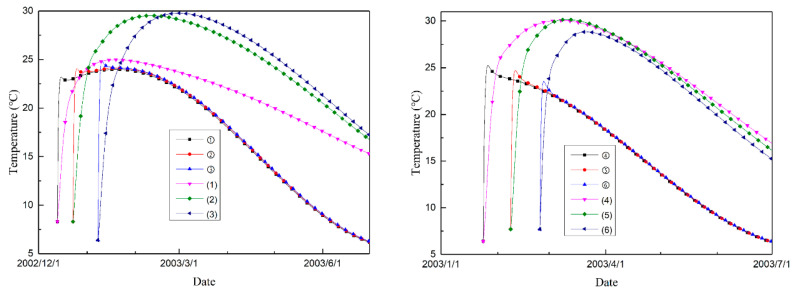
The temperature histories of different typical points.

**Figure 8:**
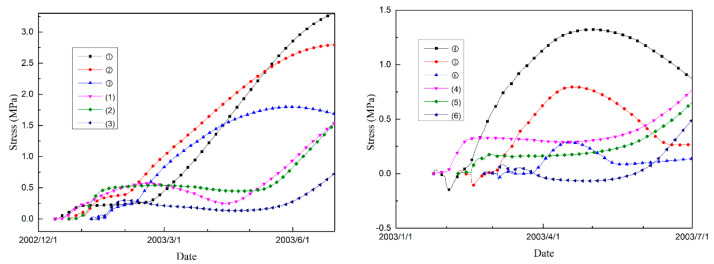
Max principal stress histories of different typical points.

Figure 7 shows the temperature history of each typical point, and Figure 8 shows the maximum principal stress histories of the typical points. The temperature procedure differences give rise to different effective stress development histories. Under normal conditions, the tensile stress increases as the temperature decreases. However, due to the expansion characteristics of MgO concrete, the autogenous volumetric strain can compensate for the shrinkage strain caused by the decrease in temperature, and the tensile stress can be reduced effectively. As shown in the stress evolution figure (Figure 8), for the typical points in both interior and exterior areas of the dam, the tensile stress does not show an upward trend for quite a few months, while the temperature decreases during that period, as shown in Figure 7.

For the nodes on the downstream profile of the dam section, the temperature rises to a certain extent and then changes with air temperature, while the temperature of the nodes on the midplane rises higher and drops more slowly due to the poor heat dissipation conditions. Therefore, for points distributed in the interior areas of the dam, the shrinkage compensating effect is superior to that of the points distributed on the exterior areas of the dam. As shown in Figure 8, for the exterior points, the period during which the tensile stress does not increase is obviously shorter than that in the interior areas of the dam. 

**Figure 9:**
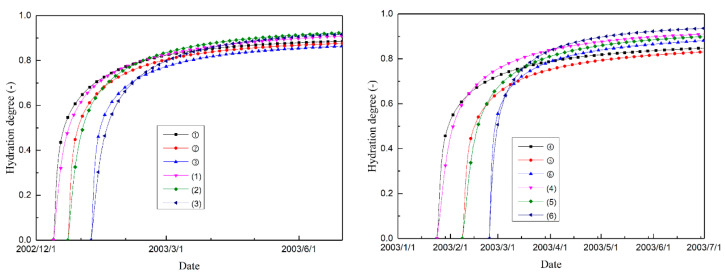
Cement hydration degree histories of different typical points.

**Figure 10:**
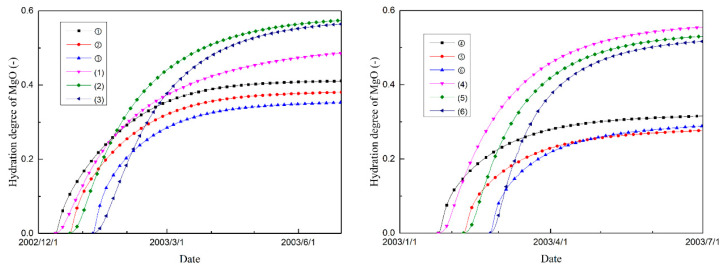
Magnesium oxide hydration degree histories of different typical points.

Figure 9 and Figure 10 show the cementitious material and magnesium oxide hydration degree histories of the selected typical points, respectively. As shown in the figures, the hydration degrees of both the cementitious material and the magnesium oxide are inhomogeneously distributed within the mass concrete structure and are affected by the distribution of temperature. The poor heat emission condition of the inner part of the dam accelerates the hydration process of the concrete in these regions. Thus, the increase in temperature (hydration of cementitious material) and expansion volumetric deformation (hydration of MgO) of the concrete on the upstream and downstream surfaces lags behind that of the inner regions.

Moreover, Figure 9 and Figure 10 show that the differences of cement hydration degree for the inner and exterior regions appear to be smaller than that of magnesium oxide hydration degree, especially at the late stage. This phenomenon may result from the activation energy of MgO hydration being larger than that of cement hydration. However, quantitative analysis for differences of internal and external expansion needs further experimental data as well as monitored data which would be scrutinized in future work.

## 5. Conclusions

In this work, a coupled thermo-chemical-mechanical model is proposed to study the expansion characteristics and mechanical responses of mass concrete structures. Focusing on the chemical-physical responses of mass concrete structures, parameter studies and numerical simulations were conducted. The major achievements are as follows:

The coupled thermo-chemical-mechanical hydration model separates the hydration degree of the cementitious material and the magnesium oxide. In addition, the heat release is determined by the hydration degree of the cementitious material, while the autogenous volumetric deformation is determined by that of magnesium oxide.

The thermo-chemical model is numerically implemented in the finite element method. In each load step of the finite element analysis, the hydration of both the cementitious material and the magnesium oxide can be updated synchronously.

The activation energy of magnesium oxide hydration, which is an important parameter in hydration kinetics, has rarely been reported in the literature. To overcome the problem, a mathematical derivation method for obtaining the activation energy is introduced.

Numerical simulations are conducted, and the results of the comparison of the experimental temperature and autogenous volumetric strain curves show that the hydration model in this work provides a relatively high accuracy.

The model is also applied in a case study on an arch dam, and the hydration degree of magnesium oxide within mass concrete, which can lead to strain compensation for shrinkage caused by temperature drop, is highlighted. The increase in temperature (hydration of cementitious material) and expansion volumetric deformation (hydration of MgO) of the concrete on the upstream and downstream surfaces lags behind that of the inner regions.

The differences of cement hydration degree for the inner and exterior regions and that of magnesium oxide hydration degree show not to be synchronized; this may be caused by the difference of cement hydration activation energy and MgO hydration activation energy. Meticulous numerical simulations and quantitatively contrastive analyses with current traditional model as well as monitoring data should be scrutinized in future work.

## Figures and Tables

**Table 1:** Component mass of oxygen magnesia concrete samples with varying fly ash contents.

	Fly Ash Content	Composition of Concrete (kg/m^3^)									Set-Retarding Superplasticizer
w/b		Water	Cement	Fly Ash	MgO	Sand	Stone (mm)				
							5–20	20–40	40–80	80–150	
0.57	30%	100	123	52	8.8	541	343	343	514	514	1.22
0.57	40%	100	105	70	8.8	541	343	343	514	514	1.22

**Table 2:** Adiabatic temperature increases of oxygen magnesia concrete samples with varying water–binder ratios.

Fly Ash Content	1 d	3 d	7 d	14 d	21 d	28 d	45 d	60 d	90 d
30%	3.63	8.64	14.24	18.81	21.06	22.40	24.15	24.95	25.80
40%	4.93	10.38	15.18	18.36	19.73	20.50	21.45	21.87	22.30

**Table 3:** Autogenous volume deformation of oxygen magnesia concrete samples at different ages with varying water–binder ratios and curing temperatures.

Fly Ash Content	Curing Temperature	Autogenous Volumetric Strain (10^−6^)									
		1 d	3 d	7 d	14 d	28 d	60 d	90 d	120 d	150 d	180 d
30%	20 °C	2.9	7.3	16.1	27.1	48.1	66.7	77	83.7	90.6	94.9
40%	20 °C	3.4	7.5	16.1	23.6	38	58.3	66.6	73.5	82.8	88.8
30%	30 °C	7.3	24.1	44.3	64	83.2	102	116.4	125.2	129.1	132
40%	30 °C	17.4	30.3	38.9	51.9	71.6	93.5	108	120	125	130.3

**Table 4:** Parameters describing the cement hydration properties.

Parameters	$\beta_{1}$ (10^6^ h^−1^)	$\beta_{2}$ (10^−2^)	$\xi_{\infty }$ (-)	$\bar{\eta }$ (-)	$Q_{\infty }$ (J kg^−1^)	$E_{a}/R$ (K)
Fly ash content 30%	1.88	5.6	1	6.6	26,313	5000
Fly ash content 40%	4.08	9.0	1	6.8	22,062	5000

**Table 5:** Parameters for describing the MgO hydration properties.

Parameters	$B_{1}$ (10^9^ h^−1^)	$B_{2}$ (10^4^)	$\zeta_{\infty }$ (-)	${\bar{\eta }}_{m}$ (-)	$E_{m}/R$ (K)	*k_a_*	*k_b_*
Fly ash content 30%	15.2	10	1	4.0	12,000	0	204
Fly ash content 40%	4.5	2.8	1	4.5	11,500	0	204

## Data Availability

Some or all data, models or code that support the findings of this study are available from the corresponding author upon reasonable request.
